# Safety of recombinant activated factor VII for bleeding use post Heart Mate III, left ventricular assist device implantation

**DOI:** 10.1016/j.jhlto.2026.100594

**Published:** 2026-05-12

**Authors:** Fahmi A. AL-Kaf, Razan AlRifaie, Hassan Songor, Mohammed Alreshidan

**Affiliations:** King Fahad Medical City - Riyadh, Kingdom of Saudi Arabia

**Keywords:** left ventricular assist device, LVAD, Heart Mate III, recombinant active factor VII, cardiac surgery bleeding, heart failure, advanced heart failure

## Abstract

**Background:**

Perioperative bleeding is a frequent high-risk complication, especially during Left Ventricular Assist Device (LVAD) implantation, and frequently necessitates reoperation. Bleeding control required many therapeutic options, and in refractory cases, recombinant activated Factor VII **(**rFVIIa) was considered one of the additional off-label options used. We report our experience with rFVIIa administration during LVAD implantation, particularly with the HeartMate III device, to assess safety outcomes, including pump thrombosis, stroke, and thromboembolic events.

**Methods:**

This is a retrospective single-center study for patients who developed refractory perioperative bleeding during LVAD, HeartMate III, implantation, and received rFVIIa due to the failure of other therapeutic options. The study was conducted from the first of December 2022–31 July 2025. The primary endpoint was a composite of pump thrombosis, stroke and thromboembolic events.

**Results:**

Among all HeartMate III LVAD implantations during the study period, eight patients received rFVIIa. There was no single recorded case of mortality, major stroke causing disability or device pump thrombosis. One patient (12.5%) experienced a minor stroke. There were a total of three patients (37.5%) identified to have a surgical source of bleeding, and two patients (25%) required operation re-exploration due to continued bleeding.

**Conclusion:**

This is a retrospective trial of a single cardiac center experience. rFVIIa use in refractory bleeding during HeartMate III LVAD implantation is safe with no significant additional risk of pump thrombosis or major stroke. A further large-scale investigation is needed to determine its safety and effectiveness.

## Introduction

Heart Failure (HF) affects more than 64 million people worldwide.^1^ Despite optimal heart failure medical, surgical and device therapy, there is still an almost 13.7% risk of developing stage D advanced heart failure, which is characterized by persistent and severe HF symptoms and significant morbidity and mortality.^2^ Left ventricular assist device (LVAD) is a durable Mechanical Circulatory Device (DMCD) which is considered one of the important options for advanced heart failure patients. LVAD therapy has evolved rapidly in recent years, with significant improvement in patient survival and quality of life.^3^

Bleeding is considered one of the most serious complications during or in the early hours post-cardiac surgery. The incidence of excessive postoperative cardiothoracic surgery bleeding approaches up to 11%.^4^ Studies on postoperative bleeding, which used a surrogate for bleeding evaluation, found that up to 10–15% patients received at least 4 Packed Red Blood Cells (PRBCs) units within the first 24 h, and up to 5% require reoperation for bleeding control.^5^, ^6^, ^7^ Furthermore, this can lead to higher perioperative risks of mortality, infection, prolonged ICU admission, vasoplegia and acute kidney injury.^8^, ^9^ Additionally, advanced heart failure patients had a higher risk of post-cardiac surgery coagulopathy bleeding, particularly in the immediate postoperative period after LVAD implantation.^10^, ^11^ This is mainly related to a congested liver and impaired function, and other comorbidities from prolonged waiting on the heart transplant list and right-sided failure.^10^, ^11^

In fact, the therapeutic management of the perioperative severe coagulopathic bleeding required a large amount of blood component transfusion to secure hemostasis. Additionally, recombinant activated Factor VII (rFVIIa) was already safely used to control refractory bleeding in patients undergoing cardiac surgery. In the presence of an artificial device like LVAD, excessive use of a large quantity of blood products, including rFVIIa, can significantly raise the risk of major serious complications, such as device pump thrombosis or major stroke. For this reason, there is a need to conduct further research to evaluate the use of specific products, such as rFVIIa, during LVAD implantation.

## Methodology

This retrospective observational study was conducted at a tertiary cardiac center. The study included all adult patients of ≥18 years old, who underwent HeartMate III LVAD implantation and subsequently received rFVIIa to manage refractory postoperative bleeding during the period of time between December 2022 and 31 July 2025. rFVIIa was administered intraoperatively at the discretion of the attending cardiac surgeon and anaesthesiologist, based on persistent bleeding unresponsive to conventional management, including transfusion of blood products and correction of coagulopathy, guided by Rotational thromboelastometry (ROTEM) for hemostasis assessment. Dosages varied and were recorded for each patient. The data were extracted from electronic health records and operative notes. Collected variables included data of patients' demographics, intraoperative calculated blood loss, transfused blood products required and total rFVIIa administered dose. Preoperative laboratory values were taken in the early morning of the day of LVAD surgery, and the postoperative values were collected immediately within the first hour of the patient's arrival to the ICU. The blood volume loss was calculated over 18 h from the patient's arrival to the ICU to the next morning of the morning shift. The final clinical safety outcomes of mortality, LVAD pump thrombosis, thromboembolic complications, minor and major stroke, RV failure, surgical source of bleeding and surgical re-exploration if required were collected. This study was approved by the institutional review board at King Fahad Medical City- Riyadh. The stroke event was according to the National Institute of Health Stroke Scale (NIHSS) as a significant neurological deficit and, often higher risk of disability with radiographic evidence of cerebral infarction or hemorrhage for the major stroke, and a Small, ischemic and non-disabling event for the minor stroke. (^12^ Moreover, any thromboembolic events other than stroke were recorded separately. The surgical cause of bleeding was defined as a bleeding resulting from a specific identifiable surgical site or technical complication (e.g., suture line, graft, or anastomosis) requiring intervention.^13^ Right ventricular failure diagnosis was based on combined clinical, hemodynamic, and echocardiographic findings, which include Hemodynamic Criteria e.g. Right Atrial Pressure (RAP/CVP) >16 mmHg AND Cardiac Index (CI) <2.2 L/min/m² despite adequate LVAD flow, End-Organ Dysfunction e.g. Worsening renal function (creatinine >2.0 mg/dL) or hepatic congestion (total bilirubin >2.0 mg/dL) and Echocardiographic Findings e.g. Severe RV dilation, severe tricuspid regurgitation (TR), and leftward interventricular septal (IVS) shift. The RV Failure Severity Grading is classified according to inotropic support and/or inhaled nitric oxide use duration, with mild when support is required for 0–7 days, moderate for a support duration between 7–14 days and severe when the need for >14 days or temporary right ventricular assist device (RVAD) support is required, regardless of the duration of support.

## Statistical analysis

All statistical analyses were performed using SPSS version 28.0 (IBM, Armonk, NY, USA). Descriptive statistics were used to summarise demographic and clinical data. The distribution of continuous variables was assessed visually using histograms. For non- symmetrically distributed variables, medians and interquartile ranges (IQRs) were reported. Paired comparisons were made using the Related-Samples Wilcoxon Signed Rank Test, with statistical significance set at a 95% confidence level (p < 0.05).

## Results

During the study period, a total of 41 patients underwent HeartMate III LVAD implantation; eight of them received rFVIIa administration. The cohort consisted predominantly of males (87.5%), with a mean age of 46.25 ± 7.81 years, a mean BMI of 24.41 ± 5.04 kg/m² and a significant portion of the patients (62.5%) were smokers (Table 1).

**Table 1:** Sociodemographic and Clinical Characteristics

Characteristic	Description	Value (N=8) - no.(%)
Age (year)	min - max	36–58
	Mean±SD	46.25±7.81
	Median (P25 - P75)	46.5 (39.5–52)
Gender	Female	1 (12.5%)
	Male	7 (87.5%)
BMI (kg/m²)*	min - max	16.7–32.8
	Mean±SD	24.41±5.04
	Median (P25 - P75)	23.45 (21.8–27.65)

Comorbidities & History	Value (N=8) - no.(%)	

Smoker	5 (62.5%)	
Diabetes Mellitus	4 (50.0%)	
Hypertensive disease	1 (12.5%)	
Atrial Fibrillation^a^	1 (12.5%)	
Ischemic Heart Disease^b^	3 (37.5%)	

Medications & Surgical History	Value (N=8) - no.(%)	

Aspirin Use^c^	3 (37.5%)	
Previous Cardiac Surgery^d^	1 (12.5%)	
INTERMACS Classification^e^:	I	1 (12.5%)
	II	1 (12.5%)
	III	6 (75.0%)

* BMI = Body Mass Index and is the weight in kilograms divided by the square of the height in meters.

a All Atrial Fibrillation patients indicated NOACs (Novel Oral Anticoagulants) used, which was held for at least 4–5 days before surgery with heparin or enoxaparin as a pre-surgical bridging therapy.

b All patients had previous percutaneous coronary intervention with drug-eluting stents.

c Aspirin was not held before surgery due to indication of single antiplatelet therapy from previous PCI.

d One patient with history of failed attempted surgical percutaneous CRT (Cardiac Resynchronization Therapy) lead implantation.

e INTERMACS Profile: Interagency Registry for Mechanically Assisted Circulatory Support classification system for heart failure severity.

Regarding comorbidities, half of the patients had diabetes mellitus (50%), but hypertension was less common, with only a single patient (12.5%). More than a third of the patients (37.5%) had ischemic cardiomyopathy with a history of previous revascularisation with percutaneous coronary intervention (PCI), while a history of Atrial Fibrillation was noted in only one patient (12.5%) (Table 1).

In terms of surgical risk profile, the majority of patients (75.0%) were classified with an INTERMACS profile III, indicating stable but inotrope-dependent status. Only one patient (12.5%) had an INTERMACS I profile; Impella CP insertion was urgently required for patient stabilisation during a critical cardiogenic shock. Another single patient with progressive decline despite initial inotrope support was established as INTERMACS II. Furthermore, three patients (37.5%) were on aspirin therapy prior to surgery as an indication of single antiplatelet therapy from previous PCI (Table 1).

All patients received rFVIIa intraoperatively with the total dose, calculated per kilogram of body weight, ranging from 26.30 to 136.36 mcg/kg, with a mean dose of 67.88 ± 35.59 mcg/kg. The median dose was 59.0 mcg/kg (IQR: 37.8–90.1 mcg/kg), indicating considerable variability in dosing requirements among patients, likely reflecting individualized treatment approaches based on the severity of bleeding and patient-specific factors.

The calculated intraoperative blood loss and blood products transfusion required demonstrates a statistically nonsignificant reduction post-rFVIIa dosage with total blood loss median from 1520 ml to 500 ml; p = 0.263, median PRBC reduced from 4 to 1.5 units (p = 0.258), Platelets units median transfusion decreased from 1.5 to 1 (p = 0.317), Fresh Frozen Plasma (FFP) median reduced from 3 to 2.5 units (p = 0.571), and the Cryoprecipitate use showed no statistically significant difference (p = 0.833) (Table 2). The ICU blood loss was calculated over 18 h from the time the patient arrived in the ICU, with a median blood loss of 1155 ml (Table 2).

**Table 2:** Intraoperative Products Required and ICU Blood Loss

Characteristic	Description	Value (N=8)		p-value
		Pre FVII dose	post FVII dose	

Tranexamic acid	min - max	1000–5186		
	Mean±SD	3033±1290.84		
	Median (P25 - P75)	3187.5 (2107–3744.5)		
PRBCs (units)	min - max	3–8	1–9	0.258
	Mean±SD	4.5±1.51	3.5±3.3	
	Median (P25 - P75)	4 (4–4.5)	1.5 (1–6.5)	
Platelet (units)	min - max	1–3	0–3	0.317
	Mean±SD	1.63±0.74	1.25±1.04	
	Median (P25 - P75)	1.5 (1−2)	1 (0.5–2)	
Fresh-frozen plasma (units)	min - max	0–10	0–7	0.571
	Mean±SD	3.88±4.09	3.13±2.75	
	Median (P25 - P75)	3 (0–7.5)	2.5 (1–5.5)	
Cryoprecipitate (units)	min - max	0–7	0–6	0.833
	Mean±SD	3.25±3.49	2.88±2.47	
	Median (P25 - P75)	3 (0–6.5)	4 (0–4.5)	
Total blood loss (ml)	min - max	640–2120	250–3200	0.263
	Mean±SD	1431.3±534.8	1072.5±1106.3	
	Median (P25 - P75)	1520 (975–1850)	500 (375–1690)	
Total FVII Doses (mcg/kg)	Min - Max	26.30–136.36		
	Mean±SD	67.88±35.59		
	Median (P25 - P75)	59.0 (37.8–90.1)		
ICU Blood Loss/18hrs(ml)*	min - max		385–2655	
	Mean±SD		1364.38±789.11	
	Median (P25 - P75)		1155 (877.5–1905)	

* ICU blood loss calculated from ICU patient arrived post-surgery to next day over 18 hrs for all patients.

Moreover, pre-and post-surgical laboratory parameters showed a significant postoperative reduction in hematocrit value from 40.75 ± 4.33–29.13 ± 5.03 (%) (p = 0.012) and in platelet count from 260.63 ± 77.9–153.38 ± 88.4 (10*3/L) (p = 0.012). Other parameters, including International Normalized Ratio (INR) and Activated Partial Thromboplastin Time (APTT), did not show statistical differences (Table 3).

**Table 3:** Laboratory Parameters

Characteristic	Description	Value (N=8)		p-value
		Pre-Surgery	Post-Surgery	
Hematocrit (%)	min - max	34–45	22–35	0.012
	Mean±SD	40.75±4.33	29.13±5.03	
	Median (P25 - P75)	42.5 (37−44)	29 (25−34)	
Platelet (10^3^/μl)	min - max	114–338	64–275	0.012
	Mean±SD	260.63±77.9	153.38±88.4	
	Median (P25 - P75)	300 (207.5–309)	127 (74.5–242.5)	
Total bilirubin (umol/l)	min - max	6.2–94	15.2–187	0.484
	Mean±SD	32.25±32.96	52.16±57.71	
	Median (P25 - P75)	18.3 (11.35–49.25)	27.45 (18.05–62.05)	
International Normalized Ratio (INR)	min - max	1.08–1.36	0.73–1.28	0.063
	Mean±SD	1.17±0.09	0.99±0.22	
	Median (P25 - P75)	1.16 (1.115–1.195)	0.97 (0.775–1.2)	
Activated partial thromboplastintime (seconds)	min - max	29.1–48.9	32.3–57.5	0.31
	Mean±SD	38.68±6.08	43.3±10.65	
	Median (P25 - P75)	38.9 (34.8–42)	39.65 (34–54.65)	
Fibrinogen level (g/L)	min - max		1.72–5.28	
	Mean±SD		3.29±1.48	
	Median (P25 - P75)		3.085 (2.34–4.245)	

*All laboratory parameters taken in the early morning time for pre-surgery results and on the first hour of arrival to ICU for post-surgery results.

Table 4 presents the outcome results among the eight patients who received rFVIIa; there was no single recorded case of mortality, major stroke causing disability or device pump thrombosis. On the other hand, there was one patient (12.5%) who experienced a minor stroke event, which was an incidental new post-operative CT brain finding of lacunar centrum semiovale hypodensity without any new related neurological deficits. A CT brain was repeated postoperatively as one of the neurological follow-up tests for chronic peripheral diabetic neuropathy required by the neurology team. Additionally, a total of three patients (37.5%) were identified to have a surgical source of bleeding and two patients (25%) required operation re-exploration due to continued blood loss. Right ventricular (RV) failure emerged as a significant early complication in this cohort, occurring in 5 out of 8 patients (62.5%) following LVAD implantation. The severity of RVF was graded from mild RVF in 2 patients (25.0% of total cohort, 40.0% of RVF cases) who required inotropic support for less than 7 days; moderate RVF in 2 patients (25.0% of total cohort, 40.0% of RVF cases) requiring inotropic support for 7–14 days; and severe RVF in 1 patient (12.5% of total cohort, 20.0% of RVF cases) who necessitated temporary right ventricular assist device (RVAD) support. The notably high incidence of RV failure in this case series may be attributed to multiple interrelated factors, including the critical preoperative status of the patients, the high product transfusion requirements, and subsequent hemodynamic compromise.

**Table 4:** Final Outcome

Characteristic	N (%)
Death	0 (0.0%)
Surgical re-exploration	2 (25.0%)
Surgical cause of bleeding*	3 (37.5%)
LVAD Pump Thrombosis	0 (0.0%)
Thromboembolism^a^	0 (0.0%)
Major stroke^b^	0 (0.0%)
Minor Stroke^c^	1 (12.5%)
Deep vein thrombosis (DVT)	0 (0.0%)
RV Failure	5 (62.5%)
RV Failure Severity^d^ (n=5)	Mild 2 (25.0%)Moderaten 2 (25.0%)Severe1 (12.5%)

* The surgical cause of bleeding was defined as a bleeding resulting from a specific identifiable surgical site or technical complication (e.g., suture line, graft, or anastomosis) requiring intervention

a Thromboembolism: defined as acute arterial blood vessel occlusion (other than CNS, Stroke) by thrombus with mostly cardio embolic in origin.

b Major Stroke definition: using the National Institute of Health Stroke Scale NIHSS, as significant neurological deficit and, often higher risk of disability.

c Minor Stroke definition: using the (NIHSS), as a Small, ischemic, non-disabling event.

d RV Failure Severity Grading (INTERMACS/MCS-ARC Criteria): Mild RVF: Inotropic support and/or inhaled nitric oxide for 0–7 days, Moderate RVF: Inotropic support and/or inhaled nitric oxide for 7–14 days and Severe RVF: Need for temporary right ventricular assist device (RVAD) support OR inotropic requirements >14 days.

## Discussion

Pump thrombosis is one of the worrying complications of LVADs. In previous generations of LVAD pumps, axial-flow of Heart Mate II and centrifugal flow of HeartWare HVAD, the risk of pump thrombosis occurs in 2–13% of adult patients.^14^, ^15^ This can lead to thromboembolic events and/or LVAD malfunction as a consequence of pump thrombosis.^16^

The HeartMate III is the latest generation and is currently the only durable LVAD re--ed by the US Food and Drug Administration.^3^This centrifugal flow pump device has important new technology design advantages that significantly improve the hemocompatibility of blood pumps, such as a magnetically levitated rotor (Full MagLev), Artificial pulse, Textured blood-contacting surfaces, significant blood-flow gaps and low shear stress.^17^ This advanced technology has an important additional benefit to the HeartMate III VAD device by significantly reducing the risk of pump thrombosis.^17^, ^18^, ^19^ The data from the MOMENTUM 3 trial indicate that LVAD implantation improves the overall survival rate 79% at 2 years and 58.4% at 5 years, with only 1.4% pump thrombosis.^18^, ^19^

The complex of exposed subendothelial Tissue Factor and native factor VII/VIIa (TF: FVIIa complex) plays a significant role in activating the extrinsic coagulation cascade, promoting clot formation, and regulating bleeding.^20^, ^21^ Recombinant FVIIa (rFVIIa), known commercially as NovoSeven, restores hemostasis through activation of Tissue Factor.^20^, ^22^ It is an administration re--ed for refractory coagulopathic bleeding post-cardiac surgery when other therapeutic options are unsuccessful.^23^, ^24^ The evidence demonstrates the benefit of rFVIIa in reducing chest tube bleeding, blood product transfusion and re-exploration rate post-refractory cardiac surgery bleeding.^25^, ^26^ The primary concern with adverse events of rFVIIa administration is the risk of thromboembolic complications. Referring to previous studies, the thromboembolic event rate in those receiving rFVIIa ranged from 5% to 25%.^26^, ^27^

rFVIIa has been employed off‑label to control severe bleeding in patients with LVADs.^28^, ^29^ The evidence outcomes varied by device type; however, its use in LVAD patients remains controversial due to significant thromboembolic risks. In the HeartMate II, reported data showed high immediate hemostatic success but a markedly increased rate of thromboembolic complications such as pump thrombosis and stroke.^30^ This data risk reveals an apparent dose‑dependent increase to 9.4 % of thromboembolic events for low‑dose (10–20 µg/kg) of rFVIIa therapy, rising to 36.7 % with high‑dose (30–70 µg/kg).^31^ The HVAD device had a higher baseline thrombosis risk, without rFVIIa therapy, which was about 5–10%.^32^ There are more limited reports for rFVIIa therapy during HVAD device implantation, with the absence of prospective or randomised trials; the available case series and registry data suggest rFVIIa is associated with a significant risk of thrombo‑embolic events.^33^

**Figure 1:**
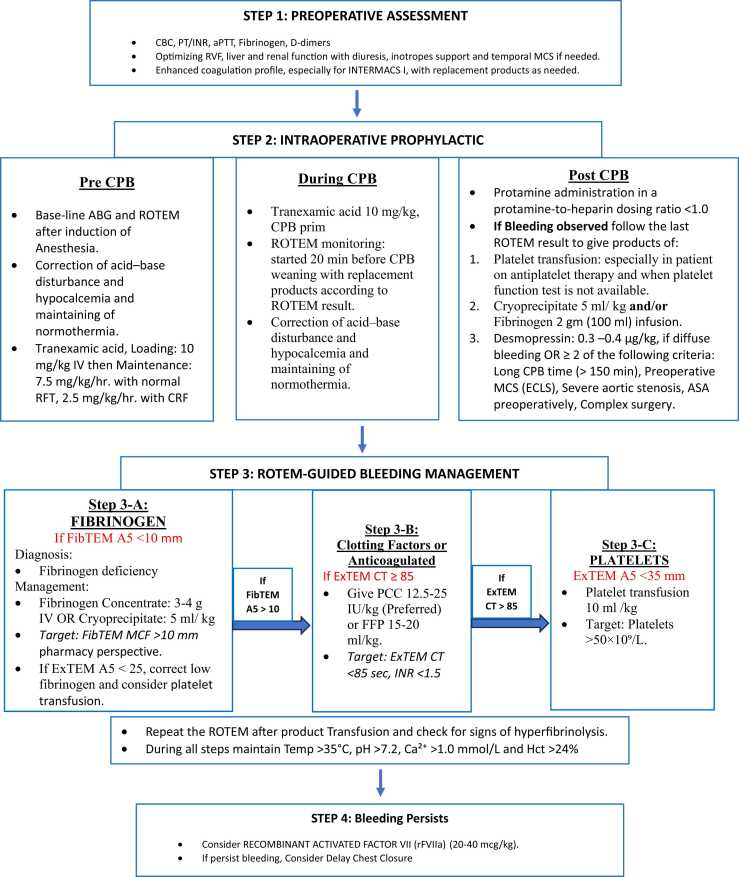
Coagulopathy Management Protocol for LVAD Surgery.

Our institutional protocol at King Fahad Medical City (KFMC) incorporates a coagulopathy management protocol for LVAD surgery in a comprehensive stepwise approach designed to minimize bleeding complications while avoiding unnecessary exposure to hemostatic rescue therapies (Figure 1). This protocol begins with preoperative optimisation of coagulation parameters, right ventricular function, and end-organ perfusion, followed by intraoperative prophylactic antifibrinolytic therapy with tranexamic acid and early ROTEM monitoring before cardiopulmonary bypass (CPB) weaning. Moreover, when persistent microvascular bleeding continues despite the initial management, the protocol escalates to ROTEM-guided targeted interventions addressing fibrinogen deficiency, platelet dysfunction, coagulation factor deficiency and metabolic optimisation with consideration of desmopressin for acquired von Willebrand disease. Finally, recombinant activated Factor VII (rFVIIa) is reserved for persistent bleeding after exhausting all previous interventions, with delayed chest closure (12–24 h) as an alternative strategy if needed.

The evidence for rFVIIa therapy during HeartMate III device implantation is limited to only one case series, with total of 4 patients.^34^ Our institution experience in HeartMate III in the current study reported the successful use of rFVIIa low or in higher doses during HeartMate III implantation supporting its safety and reflecting the technological advancements in HeartMate III device, which have generally contributed to significantly reducing the likelihood of pump thrombosis or thromboembolic events.

## Conclusion

This is a retrospective trial of a single cardiac center experience. rFVIIa use in refractory bleeding during HeartMate III LVAD implantation is safe with no significant additional risk of pump thrombosis or major stroke. A further large-scale investigation is needed to determine its safety and effectiveness.

## Contributions

All authors made significant contributions to complete the study and manuscript. Furthermore, all authors reviewed and approved this final version of the manuscript.

## Twitter

Twitter account: @dr_F_Alkaf

LVAD Bleeding Management Update: Single-center retrospective study (n=8) demonstrates safety of recombinant Factor VII use in managing bleeding during HeartMate III LVAD implantation. Results: showed zero pump thrombosis, no major strokes, and no mortality, 12.5% minor stroke. Important findings for advanced heart failure care!

#ISHLT #MCS #LVAD #HeartMate3 #Advanced HeartFailure

## Funding

None

## Declaration of Competing Interest

The authors declare that they have no known competing financial interests or personal relationships that could have appeared to influence the work reported in this paper.
